# Sci-ModoM: a quantitative database of transcriptome-wide high-throughput RNA modification sites

**DOI:** 10.1093/nar/gkae972

**Published:** 2024-11-05

**Authors:** Etienne Boileau, Harald Wilhelmi, Anne Busch, Andrea Cappannini, Andreas Hildebrand, Janusz M Bujnicki, Christoph Dieterich

**Affiliations:** Klaus Tschira Institute for Integrative Computational Cardiology, Im Neuenheimer Feld 669, 69120 Heidelberg, Germany; Department of Internal Medicine III, University Hospital Heidelberg, Im Neuenheimer Feld 410, 69120 Heidelberg, Germany; German Center for Cardiovascular Research - Partner site Heidelberg/Mannheim, Im Neuenheimer Feld 669, 69120 Heidelberg, Germany; Klaus Tschira Institute for Integrative Computational Cardiology, Im Neuenheimer Feld 669, 69120 Heidelberg, Germany; Department of Internal Medicine III, University Hospital Heidelberg, Im Neuenheimer Feld 410, 69120 Heidelberg, Germany; Institute of Computer Science, Johannes Gutenberg-University Mainz, Staudingerweg 9, 55128 Mainz, Germany; Laboratory of Bioinformatics and Protein Engineering, International Institute of Molecular and Cell Biology in Warsaw, Ul. Ks. Trojdena 4, PL-02-109 Warsaw, Poland; Institute of Computer Science, Johannes Gutenberg-University Mainz, Staudingerweg 9, 55128 Mainz, Germany; Laboratory of Bioinformatics and Protein Engineering, International Institute of Molecular and Cell Biology in Warsaw, Ul. Ks. Trojdena 4, PL-02-109 Warsaw, Poland; Klaus Tschira Institute for Integrative Computational Cardiology, Im Neuenheimer Feld 669, 69120 Heidelberg, Germany; Department of Internal Medicine III, University Hospital Heidelberg, Im Neuenheimer Feld 410, 69120 Heidelberg, Germany; German Center for Cardiovascular Research - Partner site Heidelberg/Mannheim, Im Neuenheimer Feld 669, 69120 Heidelberg, Germany

## Abstract

We present Sci-ModoM, the first next-generation RNome database offering a holistic view of the epitranscriptomic landscape. Sci-ModoM has a simple yet powerful interface, underpinned by FAIR data principles, a standardized nomenclature, and interoperable formats, fostering the use of common standards within the epitranscriptomics community. Sci-ModoM provides quantitative measurements per site and dataset, enabling users to assess confidence levels based on score, coverage, and stoichiometry. Data in Sci-ModoM is directly traceable to its sources. Users can Search and Compare over six million modifications across 156 datasets, Browse or download datasets, and retrieve metadata. A comparison tool offers a novel and unique opportunity to compare modifications site-wise across datasets, with the ability to securely upload and compare user data against latest published research. Sci-ModoM empowers researchers, including non-experts, to access a broad spectrum of recent quantitative RNA modification data, thereby enhancing the utility and impact of latest discoveries, and opening new avenues in biological and medical research.

## Graphical Abstract

**Graphical Abstract:**
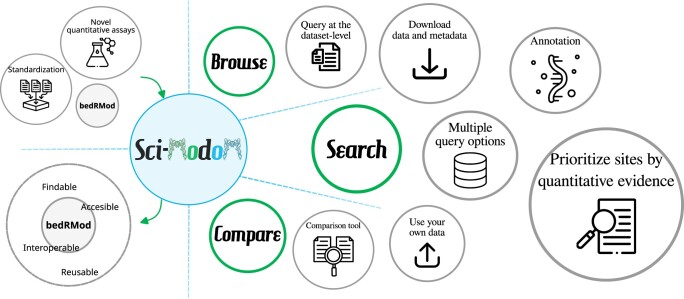

## Introduction

More than 170 distinct RNA modifications have been identified to date, with some of the most studied including methylation, pseudouridylation, and acetylation (1). Research and technology development have primarily focused on abundant mRNA modifications, such as *N*^6^-methyladenosine (m^6^A) (2,3), pseudouridine (Ψ) (4,5), *C*^5^-methylcytosine (m^5^C) (6,7), 2′-*O*-methylation (Nm) (8,9) or *N*^7^-methylguanosine (m^7^G) (10). The significance of these modifications cannot be underestimated: they affect RNA processing, stability, translation efficiency, or localization (11). Despite recent advances, particularly during the COVID-19 pandemic, our understanding of RNA modifications remains incomplete. This is primarily due to technological limitations, restricted data accessibility, and the inability to achieve long-term data usability. Variations in nomenclature and data formats across different databases continue to hinder data interoperability, often limiting the scope of research findings.

A tremendous amount of epitranscriptome sequencing data has been generated in the last few years, yet only a fraction is truly quantitative. Most databases are updated periodically and typically fail to keep up to date with latest discoveries. Technologies employing 2^nd^ and 3^rd^ generation sequencing present new opportunities for mapping and sequencing RNA and its modifications at unprecedented sensitivity and depth. Single residue resolution and quantitative, *i.e*. stoichiometric information are critical to understand how RNA modifications are regulated, and how they regulate various biological processes (12,13). But improvements in RNA modification detection has also relied on cutting-edge computational and analytic know-how that is tailored to each method and experimental protocol. Accurate quantitative information is often available for non-bioinformaticians at the expense of reproducibility.

Until now, transcriptome-wide RNA modification databases, such as REPIC (14), m6A-atlas (15), m5C-atlas (16) or RMBase (17), have hinged on evidence from limited resolution detection methods, *e.g*. RIP-seq, that involves identifying enriched regions (peaks), providing *ad hoc* quantitative profiles, or on a collection of high- and low-resolution/sensitivity data. DirectRMDB includes direct RNA sequencing data exclusively (18), while many are highly specialized databases, such as tModBase (19), m6A-atlas (15), m5C-atlas (16) or m7GHub (20). Critically, none of them provide quantitative measurements per site and per dataset that would allow to assess the confidence level of the reported modifications across datasets.

Addressing these gaps could significantly enhance the utility and impact of the accumulated knowledge on RNA modifications. A comprehensive database that consolidates and integrates high-confidence RNA modifications reported across datasets into a single, easily accessible platform is needed. Here, we present Sci-ModoM, the one-stop source for RNA modifications originating from state-of-the-art high-resolution detection methods. Its greatest appeal lies in its simplicity, yet Sci-ModoM is extremely powerful as it incorporates reliability scores and stoichiometric information per site and dataset. Sci-ModoM is entirely open source and freely available at https://scimodom.dieterichlab.org.

Sci-ModoM promotes the Findability, Accessibility, Interoperability, and Reuse (FAIR) of data by leveraging original published results, rather than re-processing raw data, by adhering to standardized naming conventions and by establishing format specifications. In particular, we present the bedRMod format (https://dieterich-lab.github.io/scimodom/bedrmod.html), an extension of the ENCODE bedMethyl format (BED9+2) for RNA modification, compatible with genome browsers. The format specifications incorporate uniformized scores, coverage and modification frequency information, allowing users to assess the confidence level of the reported modifications across datasets. The bedRMod format captures metadata, including persistent identifiers, ensuring minimal data lineage and traceability. The data in Sci-ModoM is independent of specialized processing; it relies solely on the authors’ published results and is accessible in a human-readable, interoperable format. It is the first RNA modification database to incorporate these features, aiming to engage the entire epitranscriptomics community.

A standardized nomenclature is used in Sci-ModoM to describe: modifications, using the MODOMICS (21) modification short name; organism, using the NCBI Taxonomic identifier (22); and detection technologies, using a classification based on the underlying assay, *e.g*. chemical- or enzyme/protein-assisted sequencing, and native RNA or cDNA sequencing (23). Finally, by implementing continuous data integration, Sci-ModoM enhances the relevance and timeliness of new discoveries. Thus, it has the potential to extend, in a few years only, to all of the relevant public epitranscriptome data.

**Figure 1.**
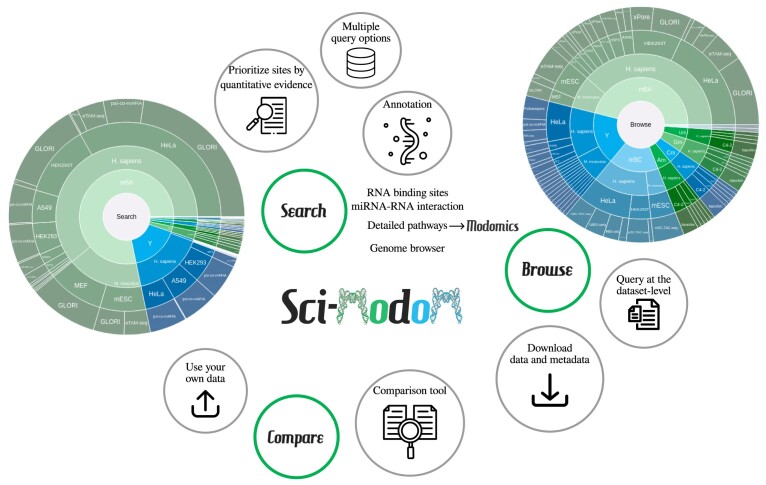
Overview of Sci-ModoM. Search over 6 M reported modifications using multiple query options (modification, organism, technology, gene, genomic features, gene biotypes), prioritize results by quantitative evidence, or recover context and interactome-relevant information. Browse through 156 datasets at the metadata-level, filtering by modification, organism, or technology, and download data in a standardized format (bedRMod). Compare datasets to one another by finding sites common to multiple datasets, sites that are close, but not intersecting, or sites only found in a group of datasets. Use available datasets, or upload your own data to compare it against database records, without registration or login.

As of August 2024, Sci-ModoM consisted of over six million quantitative sites reported across 156 datasets, for various cell types or tissues. Data is accessible via a learnable web interface to Search and Compare modifications site-wise across datasets, to recover annotation, post-transcriptional regulatory mechanisms such as RNA binding proteins or miRNAs, and to Browse through or download datasets and retrieve metadata (Figure 1).

## Materials and methods

### Data acquisition

To be included into the database, published data must, minimally, provide stoichiometric information, or frequency of modification per-site, either directly, or in a way that can be easily derived from the data only. Ideally, coverage or number of reads at a given position, and *P* values (score) should be provided (see Supplementary Methods and Supplementary Figure S1 for details and references).

### Data format

Data accessibility and interoperability is a major hurdle to be overcome by the epitranscriptomics community. To facilitate data sharing, data reuse and subsequent data mining and discovery, Sci-ModoM is promoting the implementation of common standards. With this aim, we have extended the ENCODE bedMethyl format (BED9+2), which we refer to as the bedRMod format (https://dieterich-lab.github.io/scimodom/bedrmod.html, and Supplementary Methods). This format is also compatible with the extended bedMethyl format proposed by Oxford Nanopore Technologies (https://nanoporetech.github.io/modkit).

### Annotation of modification sites

All gene assembly and annotation information were downloaded from Ensembl release 110 (24), and used to annotate datasets. Modification sites were classified into genomic regions (5-′UTR, 3-′UTR, CDS, exon, intron and intergenic) and gene biotypes. miRNA-target interactions for human and mouse were downloaded from TargetScan (25), converted to BED6, and lifted over to the latest assembly (GRCh38 and GRCm39). RNA-binding proteins (RBPs) motifs and target sites were downloaded from the oRNAment database (26), wrangled to BED6 and annotated with strand information. Data that could not be annotated were discarded. Only motif matches for the top 30^th^ percentile were kept based on the matrix similarity score (MSS). The mouse data were lifted over to GRCm39.

### Database and web interface implementation

Sci-ModoM is a multi-container application orchestrated in a rootless architecture and enhanced security environment using Podman (https://podman.io). Sci-ModoM deployment is automated in a fully reproducible manner. All datasets were processed and stored in a MariaDB (https://mariadb.org) database. The database query and REST-API backend were developed using the SQLAlchemy Database Toolkit for Python (https://www.sqlalchemy.org) and Flask (https://flask.palletsprojects.com). The API is versioned and the version is included in the API’s URL. Database migration is handled using Alembic (https://alembic.sqlalchemy.org), providing non-linear, dependency-graph versioning. The client interface was built using Vue.js (https://vuejs.org) and the PrimeVue Tailwind CSS based UI component library (https://tailwind.primevue.org). For details of the infrastructure, database schema, and software versions, consult the source repository (https://github.com/dieterich-lab/scimodom).

## Database content and web interface

### Database content

As of August 2024, Sci-ModoM consisted of 6 004 392 quantitative sites reported across 156 datasets, chiefly among human and mouse, for various cell types or tissues (Figure 1). Modifications included whole-transcriptome sequencing m^6^A, m^5^C, Ψ, 2′-*O*-Me, and m^7^G, and all sites were annotated using Ensembl release 110.

### Web interface and usage

Sci-ModoM provides an intuitive and learnable web interface divided into three major endpoints: Search, Browse, and Compare, each complemented by documentation and how-tos (Figure 1). Sci-ModoM does not require any login or registration, but optional sign-up and login functionalities are available, with access management and data upload (consult https://scimodom.dieterichlab.org/documentation/management for more information).

#### Search

**Figure 2.**
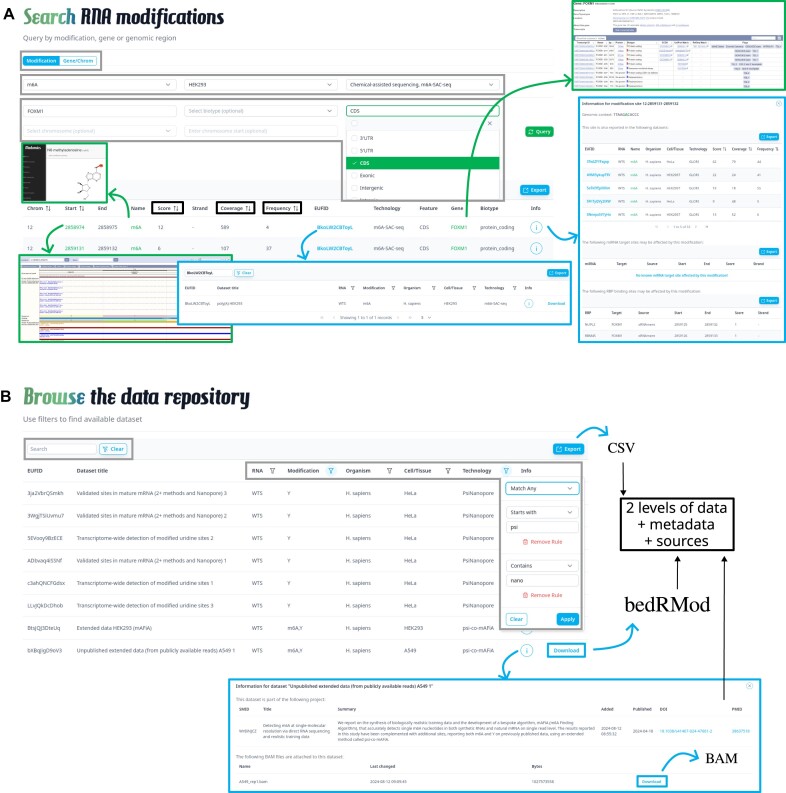
Illustration of Search and Browse queries. (**A**) The Search view allows to perform queries with multiple options (modification, organism, technology, gene, genomic features, gene biotypes), to prioritize results by quantitative evidence, to visualize modifications, recover pathways, detailed information on residues, context and interactome-relevant information. (**B**) The Browse view allows to query metadata and to download datasets in bedRMod format.

modifications site-wise across whole transcriptomes, stratified based on a choice of RNA modification, organism, and detection technologies, or by gene or genomic region (Figure 2A and Supplementary Figure S2). A search query can always be narrowed down by selecting a gene or a genomic region (chromosome and coordinates), sites pertaining to certain gene biotypes and/or associated with genomic features such as exons, introns, *etc*. (Figure 2A, grey boxes). The results table links every reported site to its genomic position on the Ensembl genome browser, each annotated site to the respective Ensembl gene table (24), and each modification to its MODOMICS reference (Figure 2A green boxes/links). Dataset information is accessible via a link to the Browse endpoint (Figure 2A, blue box/link). All records can be exported to CSV. Most importantly, the full results table can be sorted based on modification stoichiometry, or frequency, coverage, and/or score, allowing users to prioritize reported sites in a reproducible and quantitative manner (Figure 2A, black boxes). By clicking on the info button, site-specific information is shown, including genomic sequence context, datasets that report this site, including dataset-specific frequency, coverage and score, as well as miRNA target and RBP binding sites that may be affected by the modification (Figure 2A, rightmost blue box). Each table can also be exported to CSV.

#### Browse

the database to find datasets at the metadata-level. The Browse page displays in tabular form all datasets in Sci-ModoM, searchable globally, or using specific criteria such as RNA type, modification, organism, cell type or tissue, and technology (Figure 2B grey boxes). The full list of datasets and their metadata can be exported to CSV. By clicking on the info button, project-related information is shown, including data sources, as well as BAM file attachments (Figure 2B, blue box). The possibility to attach BAM files to datasets allow users to provide and retrieve intermediate information such as read-level modification information, before aggregation into bedRMod format. Most importantly, each dataset can be downloaded in bedRMod format.

#### Compare

**Figure 3.**
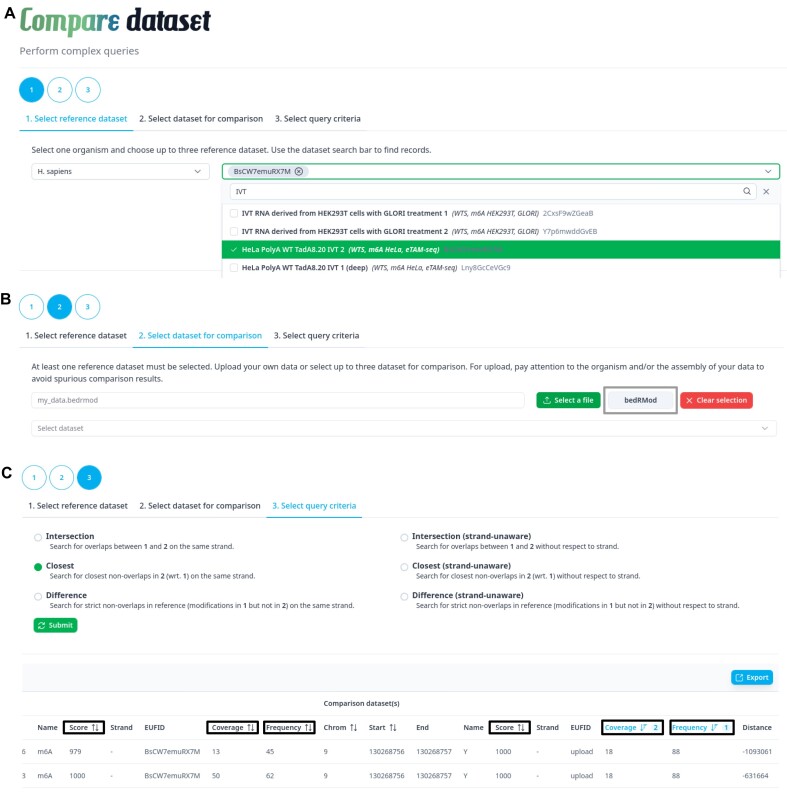
Compare datasets to one another by finding intersection or difference between datasets at the level of reported sites. It is also possible to find modification sites in a given dataset that are close to, but not intersecting with sites in other datasets. (**A**) First select a given organism, then select up to three reference datasets. (**B**) Second, select up to three datasets for comparison, or use your own data (bedRMod or BED format). (**C**) Select the operation, and prioritize results.

datasets at the records-level. The comparison tool offers a novel and unique opportunity to compare modifications site-wise across datasets, to search for overlaps or intersections (sites common to multiple datasets), closest non-overlaps (sites close to, but not intersecting), or strict non-overlaps (sites only found in a group of datasets) (Figure 3). First, select a species and up to three available reference datasets (Figure 3A). Second, select datasets to compare, or upload data to compare it against selected reference datasets (Figure 3B). Users can freely upload data in BED6 or bedRMod format without registration, for the single purpose of comparing their data against the database. Data is securely handled and is automatically deleted afterwards. Finally, select the operation to perform on the datasets selected in the previous steps (Figure 3C). This allows to find out, in a few click, the agreement or disagreement between datasets, and to discover sites close to one another across datasets, *e.g*. possible interaction between different modifications such as m^6^A and Ψ. The results table can be sorted by frequency, coverage, and/or score, based on reference or compared (or uploaded) datasets (Figure 3C black boxes). All results can be downloaded as a CSV table for further data mining.

## Results

### Use case: the presence of m^5^C in mRNA of putative m^5^C-RNA-binding proteins

ALYREF was the first proposed ‘reader’ of m^5^C in mRNA. Yang *et al.* conducted RNA pull-down experiments using m^5^C-modified RNA oligos and their unmodified counterparts, and through mass spectrometry, they identified ALYREF as the most enriched protein in the m^5^C-pull-down fraction (27). As a crucial component of the RNA Transcription and Export (TREX) complex, the authors suggested that mRNA nuclear export defects observed following the knockdown of the methyltransferase NSUN2 could be due to a reduced affinity between ALYREF and its mRNA targets. A Sci-ModoM search for m^5^C in HeLa cells using all available assays yields 10 m^5^C mRNA sites for the ALYREF locus from 3 independent studies (6,7,28) (Supplementary Figure S3). However, all reported modification frequencies are low and one study does not report any change in modification frequency upon knockdown of NSUN2 and NSUN6. To the contrary, the mRNA of another well described potential m^5^C reader protein, YBX1 (29), contains only one reported m^5^C site (30), which is not validated by at least two studies. As an intuitive first result, we investigated whether the mRNAs of two candidate m^5^C-RNA-binding proteins, ALYREF and YBX1, contain m^5^C sites. For the latter, we found no evidence. For ALYREF, we could identify one common site (17:81,888,298-81,888,299), albeit at low modification frequency ($< 10\%$).

### Use case: comparison of complementary assay technologies in the same cell line

**Figure 4.**
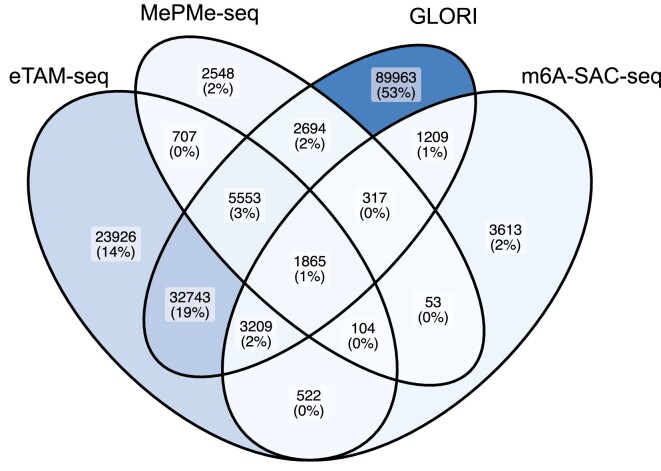
Venn diagram for a comparison of four assay technologies for m^6^A detection in HeLa cells. The total number of sites amounts to 68 629 for eTAM, 13 841 for MePMe-seq, 137 553 for GLORI and 10 892 for m6A-SAC-seq.

The sunburst plot on the Sci-ModoM title page shows that 4 complementary assay technologies for m^6^A (only) detection are available in HeLa cells: eTAM-seq, GLORI, MePMe-seq, and m6A-SAC-seq. At the time of writing, these data were distributed across 31 data sets (Supplementary Results and Supplementary Figure S4). We have exported the content of the Browse table (Export), and selected one data set per assay for untreated/wildtype conditions (EUFID: 93arJtwJmuS9, UKen6WEino9G, VL7zkHvkfkoB and aJksAshEgQ8U). A comparison for single residue overlap with bedtools (31) is shown in Figure 4. We acknowledge potential differences in sensitivity and focus on the sites that were reported by at least 3 out of 4 assays: 1865 + 317 + 5553 + 3209 + 104 = 11 048 sites. For example, for the MePMe-seq data, 79.1% of all reported MePMe-seq sites (13 841) were found in at least 2 additional assays. With this small use case, we have demonstrated how one could prioritize m^6^A sites for a given cell type. We also note that every site reported in the Search table has an associated information page, which is accessible via the "info" button, that lists all datasets in which this site has been reported.

### Use case: overlap of RNA-binding protein target sites with RNA modifications

Fagre and Gilbert discuss the relevance of RNA modifications on the interaction of RNA-binding proteins (RBPs) with RNA (32). We were inspired by this review to use the Compare tool of Sci-ModoM to intersect RBP eCLIP data originating from HepG2 cells with m^7^G, m^6^A and Nm modification site records for the same cell type. According to Zhao *et al.* (33), QKI binds to internal m^7^G sites, but only with specific isoforms QKI6 and 7, which are unfortunately not available in the ENCORE database. However, we could identify eCLIP data for RNA-binding proteins IGFBP1 and IGFBP3, which are thought to preferentially bind to m6A-modified RNAs (34). We retrieved and formatted the corresponding BED6 files for IGFBP1 and IGFBP3 and used them in searches across m^7^G, m^6^A and Nm modification site records (EUFID: YkSG92k4EHDM, nfCHyrYzE6fB and GFGhkyJXxJSx). We used the strand-specific intersect option to overlap RBP binding sites with the respective modification tracks and found 1,550 and 1,591 overlaps for the two proteins, respectively (Supplementary Results and Supplementary Figure 5). Please note that eCLIP peaks may extend over larger intervals: median for IGF2BP1 peak width is 54 and 55 bp for IGF2BP3.

Table 1 shows the results of these comparisons. We performed a Fisher’s exact test on the respective 2x2 count tables using the universe of IGF2BP1 or IGF2BP3 binding sites and all respective unique modification sites (Nm, m^6^A and m^7^G), see Supplementary Table S1. Strikingly, we could not confirm an enrichment of neither IGF2BP1 (OR = 0.79) nor IGF2BP3 binding sites (OR = 0.48) on reported m6A residues in HepG2 cells. However, the opposite was true for Gm residues, where we saw the highest significant ORs of 1.58 and 1.56, respectively. We acknowledge that the comparison is purely based on site counts. We cannot rule out that these results are prone to dataset-specific biases, but nevertheless this showcase the utility of Sci-ModoM with this particular scientific question.

**Table 1.** Overlap of mRNA modification data with ENCORE eCLIP tracks from IGF2BP1 and 3

Modification	Overlap with IGFBP1	Overlap with IGFBP3	Total
Am	363	338	13 549
Cm	298	388	10 936
Gm	489	507	18 947
Um	196	227	8010
m^6^A	191	125	11 270
m^7^G	13	6	801
**Unique**	1526	1591	

## Discussion and conclusions

By standardizing and integrating high-throughput epitranscriptome sequencing data without re-analysis, Sci-ModoM offers a unified repository for researchers to access and reuse the most recent data from novel assays. Sci-ModoM enables quantitative investigation of over six million sites across 156 datasets, spanning 15 high-resolution technologies. Users can securely upload, compare, and validate their own data against the latest state-of-the-art datasets in just a few clicks.

Many RNA modification databases have been developed to catalog and analyze RNA modifications across species and their roles in health and disease. Until now, transcriptome-wide RNA modification databases, such as REPIC (14), m6A-atlas (15), m5C-atlas (16) or RMBase (17) have relied on unequal data quality (high/low resolution) and customized data processing pipelines, sometimes resulting in poor comparability between databases. Others, such as m7GHub (20), OpenAc4C (http://www.rnamd.org/ac4cportal), as well as m6A-atlas (15) and m5C-atlas (16), tModBase (19) and DirecRMDB (18), are databases specializing in one modification type, one RNA type, or one detection methodology, respectively. Databases such as RNAmod (35), RM2Target (36), or RMVar (37) and RMDisease (38) focus on annotation of mRNA modifications, on associations between modification writers, erasers and readers with their targets, or on genetic variants affecting modifications, respectively. But critically, it is not possible for a user to easily compare the original data (*e.g*. published data) with that obtained from the database. Sci-ModoM enhances these efforts with its comprehensive, quantitative approach.

Sci-ModoM, a play on words with MODOMICS (21), serves as a one-stop source for transcriptome-wide high-throughput data; MODOMICS details individual modified ribonucleosides, their structures, biosynthetic pathways and enzymes. The two databases are different but synergistic, with Sci-ModoM linking to MODOMICS, and future plans focus on reciprocal links in MODOMICS’ next update.

Developed as a comprehensive FAIR meta-database, Sci-ModoM unifies data from scattered sources into a centralized repository, allowing users to access data in a standardized and interoperable format. By avoiding complex data processing, by relating data to its original sources, and by promoting common standards, Sci-ModoM encourages the participation of the epitranscriptomics community. Importantly, users, not maintainers, can now independently assess the reliability of reported sites using scores, coverage, and stoichiometry.

## Future directions

While Sci-ModoM is well positioned to grow towards becoming the broadest repository of epitranscriptomics data worldwide, it is not yet comprehensive. In a first stage, Sci-ModoM has focused on integrating whole-transcriptome sequencing data (mostly mRNA and lncRNA) originating from novel assays, from human and mouse. Extending Sci-ModoM to a wide-ranging number of species, and other RNA types, or allowing a finer-grain classification of RNA types, is an obvious next step. There is also an interest in adding RNA processing events. Our current understanding of RNA maturation and degradation is constantly enriched by fundamental discoveries, but there is to date no single database exploring the interplay between modification and processing. The bedRMod format is a good starting point to explore this avenue.

## Supplementary Material

gkae972_Supplemental_Files

## Data Availability

No new data were generated in support of this research. The Sci-ModoM database is freely available at https://scimodom.dieterichlab.org. The Sci-ModoM source code is an open source software freely available at https://github.com/dieterich-lab/scimodom under the GNU Affero General Public Licence (AGPL) version 3 and https://doi.org/10.5281/zenodo.13911907. The bedRMod format specification is described at https://dieterich-lab.github.io/scimodom/bedrmod.html.
