# Supplementary material for: Sci-ModoM: a quantitative database of transcriptome-wide high-throughput RNA modification sites
Source: Nucleic Acids Res. 2024 Nov 5;53(D1):D310–7. doi: 10.1093/nar/gkae972 (PMC11701610; doi:10.1093/nar/gkae972)
Supplement: gkae972_Supplemental_Files [file gkae972_supplemental_files.zip › supplementary_material.pdf]

Supplementary Material
Sci-ModoM: a quantitative database of transcriptome-wide high-throughput RNA modification sites

Etienne Boileau Harald Wilhelmi Anne Busch
Andrea Cappannini Andreas Hildebrand Janusz M. Bujnicki
and Christoph Dieterich

October 1, 2024

1 MATERIALS AND METHODS

1.1 Data acquisition

Sci-Modom entirely relies on data reuse from the authors' published results; there is no specialized data processing pipeline. To be included into the database, published data must, minimally, provide stoichiometric information, or frequency of modification per-site, either directly, or in a way that can be easily derived from the data only. Ideally, coverage, or number of reads at a given position, and p values (score) should be provided.

Based on these criteria, high-throughput m5C-TAC-seq [1], BID-seq [2], UBS-seq [3], RBS-seq [4], GLORI [5], MePMe-seq [6], m6A-SAC-seq [7], NanoNm [8], PsiNanopore [9], Nm-Mut-seq [10], eTAM-seq [11], xPore [12], PRAISE [13], mAFiA [14], and m7G-seq [15] datasets were included.

Published results were either retrieved from the Gene Expression Omnibus (GEO) [16] and/or the supplementary data associated with each original reference. Whole-transcriptome sequencing data (mostly mRNAs and ncRNAs) were converted to bedRMod, lifted over using CrossMap [17], where relevant, and integrated in the database, see Supplementary Figure 1.

1.2 Data format

The bedRMod file is a tabulated count of base modifications from every sequencing read over each reference genomic position or modification site. It is a convenient representation of the information stored in the MM/ML tags in BAM alignment files, *cf.* SAM/BAM and related specifications. It includes a header section with metadata information. The header also allows users to record information to facilitate minimal data lineage and traceability. The data section generally adheres to the bedMethyl definition, except that the name (4th column) must conform to the MODOMICS nomenclature [18] for the modification short name, and the score (5th column) is defined as $round(-log_{10}(\text{p value}))$, where p value is calculated from a statistical test. For more information, consult the bedRMod format specifications.

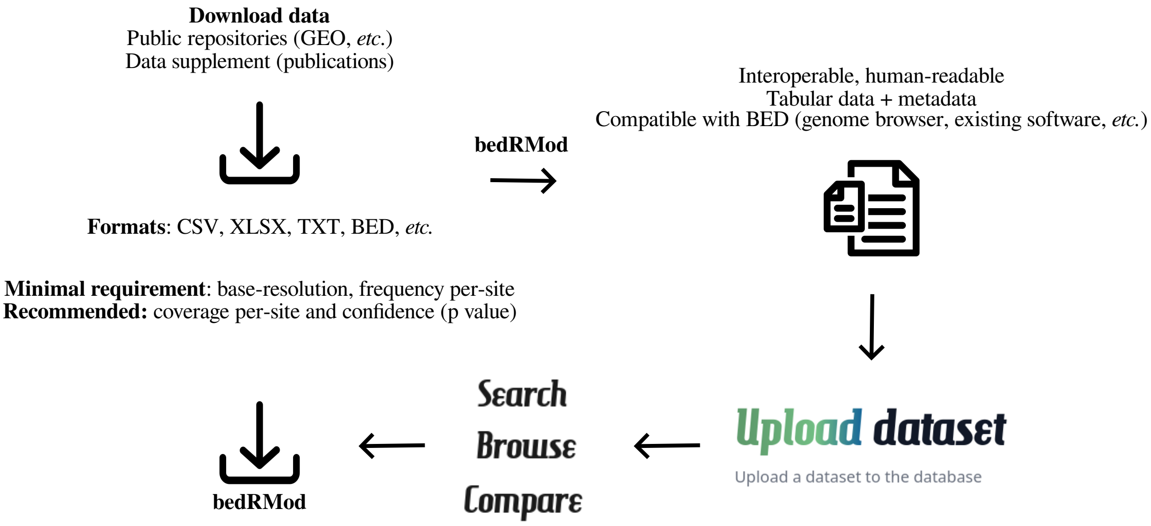

Supplementary Figure 1: Data acquisition and format. Published data must provide frequency of modification per-site, either directly, or in a way that can be easily derived from the data only, without specialized treatment or computational pipeline. bedRMod-formatted data, including metadata, is ingested into the database, via the web interface, or via a command-line interface, *cf.* Sci-ModoM server (Flask CLI). Where relevant, data is lifted over automatically (to GRCh38 or GRCm39). Each dataset is now accessible and reusable in a simple, standardized format, and carry minimal metadata information.

2 DATABASE CONTENT AND WEB INTERFACE

2.1 Web interface and usage

A toggle button allows to query by modification, organism, and technology, *cf.* manuscript Figure 2, or by gene (or genomic region), see Supplementary Figure 2.

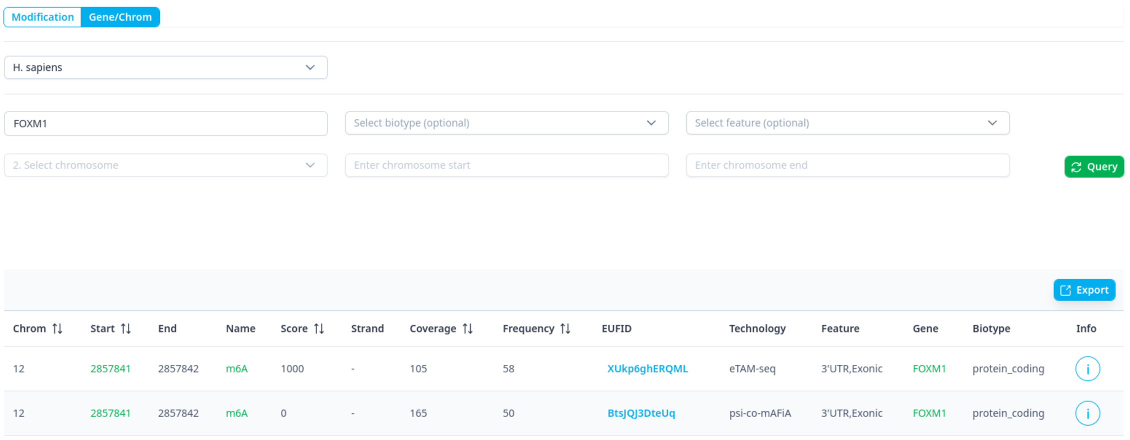

Supplementary Figure 2: The Search view allows to perform queries with multiple options, to prioritize results by quantitative evidence, to visualize modifications, recover pathways, detailed information on residues, context, and interactome-relevant information. The gene search option is shown above, allowing users to query all modifications within a given gene or genomic region.

3 RESULTS

3.1 Use case: The presence of m5C in mRNA of putative m5C-RNA-binding proteins

In this use case, we study the m5C modification status of mRNAs of candidate m5C-RNA binding proteins. We selected two examples from literature: ALYREF [19] and YBX1 [20]. The respective search is conducted through Sci-ModoM's Search tab. Subsequent selection steps are as follows: Modification: **m5C** → Species: Homo sapiens / **HeLa** cell line → Assays : **All** → Gene name: **ALYREF**, see Supplementary Figure 3.

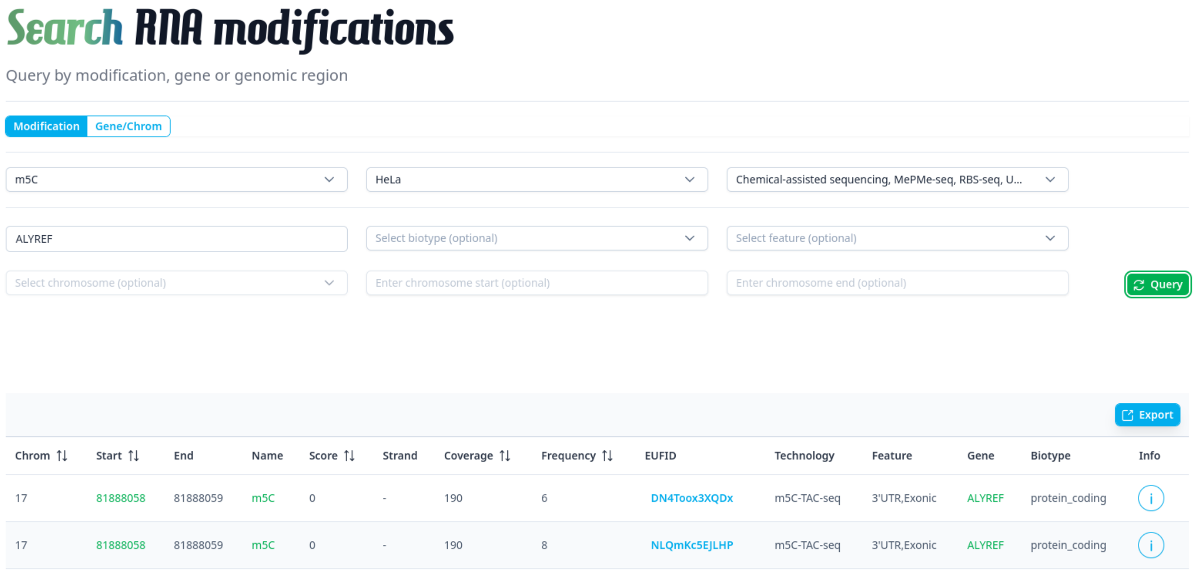

Supplementary Figure 3: Screenshot of ALYREF search. An intuitive user interface allows to select the modification type of interest in the corresponding biological system for either all or a particular gene locus.

3.2 Use case: Comparison of complementary assay technologies in the same cell line

In the second use case, we explore the overlap between different assay technologies for a given modification. Supplementary Figure 4 represents all key steps for a search of all m6A assays in HeLa cells.

The search result can be exported by the respective top right button and loaded in a spreadsheet application. We have stored the query result as Supplementary File 1: *scimodom_browse_2024-08-13T090757.xlsx*. From the entire set of 31 data sets, we selected one data set per assay for untreated/wildtype conditions (EUFID: 93arJtwJmuS9, UKen6WEino9G, VL7zkHvkfkoB and aJksAshEgQ8U). We downloaded bedRmod files for all of the respective data sets, sorted its content by genomic coordinates and removed the header for further use by bedtools [21]. We used the `bedtools multiinter` subcommand to identify common sites among multiple BED files.

Subsequently, we generated a Venn diagram with the `ggVennDiagram` package in the R language (version 4.3.2). The sorted BED files are made available as Supplementary Files 2-5:

1. sorted_GLORI1.bed (137,675 records, SFile 2)
2. sorted_MePme1.bed (13,878 records, SFile 3)

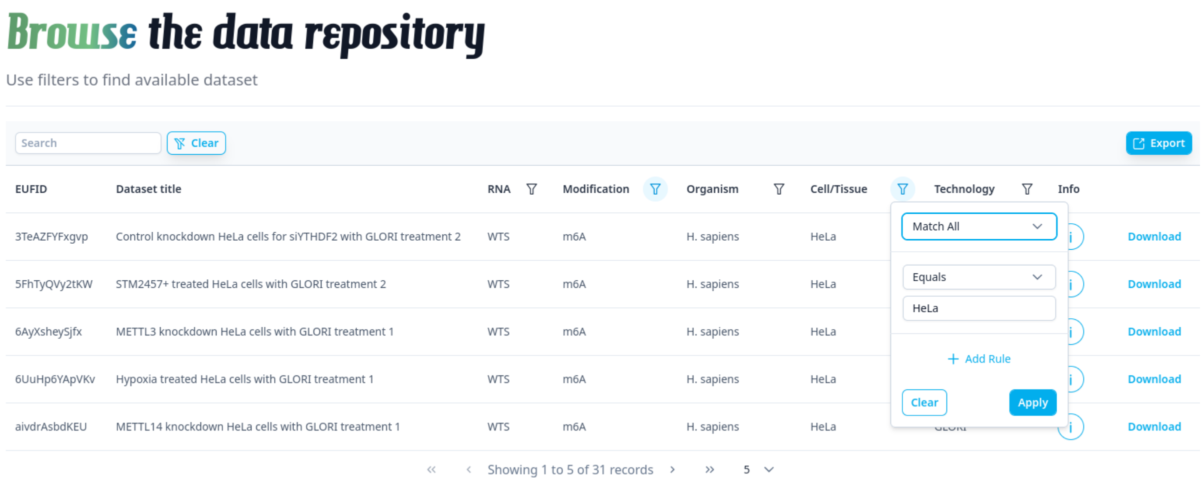

Supplementary Figure 4: Data set search for m6A assays in HeLa cells via Browse Tab. The search was restricted on the modification type: **m6A** and Cell/Tissue type: **HeLa** - see selection box.

3. sorted_eTAM1.bed (69,834 records, SFile 4)

4. sorted_m6A-SAC.bed (10,892 records, SFile 5)

3.3 Use case: Overlap of RNA-binding protein target sites with RNA modifications

The last use case employs the Compare Tab functionality with integrated file upload. We have obtained to BED6 files that comprise target sites i.e. eCLIP peaks of RNA binding proteins IGF2BP1 and IGF2BP3. The corresponding data originate from the ENCORE subsection of the ENCODE consortium. The corresponding files are

1. IGF2BP1_HepG2_GRCh38_reformat.bed (4459 records, SFile 6)

2. IGF2BP3_HepG2_GRCh38_reformat.bed (2247 records, SFile 7)

BED6 file format conventions are strictly enforced. For example the score column (5th) must be an integer between 0-1000.

The eCLIP experiments have been performed in the HepG2 cell line. Evidently, we look for available RNA modification tracks in HepG2 cell lines first. Through the Browse Tab, we identified 6 matching data sets from which we chose 3 (upper limit of Compare Function): m7G-seq (EUFID: GFGhkyJXxJSx), m6A-SAC-seq (EUFID: nfCHyrYzE6fB) and NanoNm (EUFID: YkSG92k4EHDM). Subsequently, two independent searches were performed. One for each RBP separately. Supplementary Figure 5 highlights all relevant steps in this procedure.

The result of this comparison is then further processed in the R language to produce 2x2 contingency tables. See Supplementary Table 1 (panel A) for an example. All combinations of RBP × modification were tested (Supplementary Table 1, panel B).

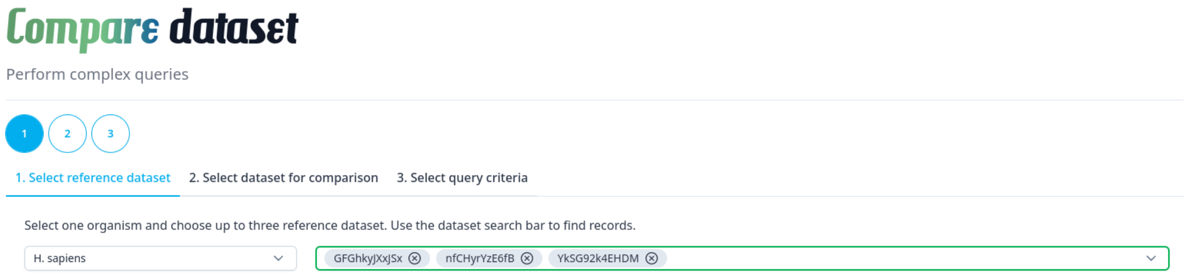

(a) Step 1. Select up to three reference data sets.

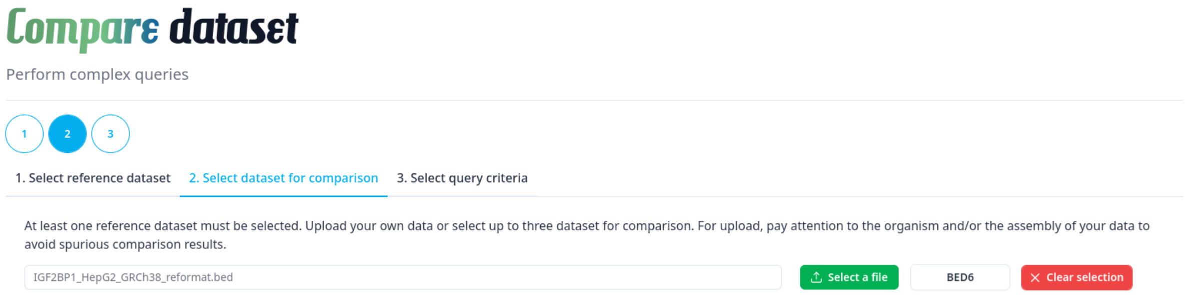

(b) Step 2. Select an external data set for upload and comparison.

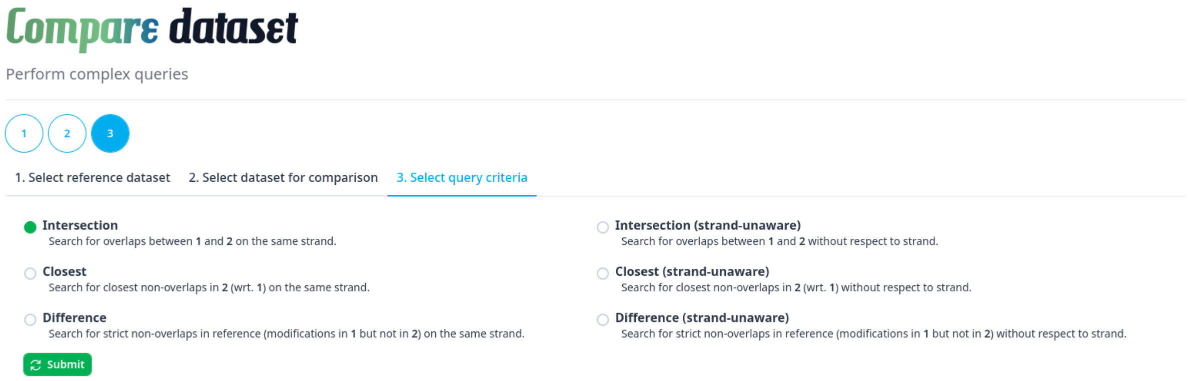

(c) Step 3. Choose the mode of comparison, which is a strand-specific intersection operation for this particular use case.

Supplementary Figure 5: Comparing RBP binding sites with RNA modification tracks.

Panel A

2x2 table	RBP bound	RBP not bound
MOI	a	b
Other	c	d

Panel B

**Modification**	**IGFBP1**		**IGFBP3**	
	OR	p-value	OR	p-value
Am	1.46	2.38e-09	1.28	0.0001231
Cm	1.39	7.588e-07	1.91	< 2.2e-16
Gm	1.56	4.132e-15	1.58	2.456e-16
Um	1.157	0.06036	1.33	0.0001275
m6A	0.794	0.002775	0.476	< 2.2e-16
m7G	0.669	0.1633	0.295	0.0005056

Supplementary Table 1: Panel A. Example of a contingency table for Fisher's exact test. Number of RNA modification sites, which fulfill criteria as depicted on rows and columns. The odds ratio is computed as $(a * d)/(b * c)$. MOI=Modification of interest. Panel B. Overlap of mRNA modification data with ENCORE eCLIP tracks from IGF2BP1 and 3. OR: odds ratio, p-value: two-sided Fisher's exact test.

References

[1] Lu, L., Zhang, X., Zhou, Y., Shi, Z., Xie, X., Zhang, X., Gao, L., Fu, A., Liu, C., He, B., Xiong, X., Yin, Y., Wang, Q., Yi, C., and Li, X. (July, 2024) Base-resolution m5C profiling across the mammalian transcriptome by bisulfite-free enzyme-assisted chemical labeling approach. *Molecular Cell,* pp. S1097–2765(24)00528–8.

[2] Dai, Q., Zhang, L.-S., Sun, H.-L., Pajdzik, K., Yang, L., Ye, C., Ju, C.-W., Liu, S., Wang, Y., Zheng, Z., Zhang, L., Harada, B. T., Dou, X., Irkliyenko, I., Feng, X., Zhang, W., Pan, T., and He, C. (March, 2023) Quantitative sequencing using BID-seq uncovers abundant pseudouridines in mammalian mRNA at base resolution.. *Nature biotechnology,* **41**, 344–354.

[3] Dai, Q., Ye, C., Irkliyenko, I., Wang, Y., Sun, H.-L., Gao, Y., Liu, Y., Beadell, A., Perea, J., Goel, A., and He, C. (January, 2024) Ultrafast bisulfite sequencing detection of 5-methylcytosine in DNA and RNA. *Nature Biotechnology,*.

[4] Khoddami, V., Yerra, A., Mosbruger, T. L., Fleming, A. M., Burrows, C. J., and Cairns, B. R. (2019) Transcriptome-wide profiling of multiple RNA modifications simultaneously at single-base resolution. *Proceedings of the National Academy of Sciences,* **116**(14), 6784–6789.

[5] Liu, C., Sun, H., Yi, Y., Shen, W., Li, K., Xiao, Y., Li, F., Li, Y., Hou, Y., Lu, B., Liu, W., Meng, H., Peng, J., Yi, C., and Wang, J. (March, 2023) Absolute quantification of single-base m, javax.xml.bind.JAXBElement@1acf3247, A methylation in the mammalian transcriptome using GLORI.. *Nature biotechnology,* **41**, 355–366.

[6] Hartstock, K., Kueck, N. A., Spacek, P., Ovcharenko, A., Hüwel, S., Cornelissen, N. V., Bollu, A., Dieterich, C., and Rentmeister, A. (Nov, 2023) MePMe-seq: antibody-free simultaneous m6A and m5C mapping in mRNA by metabolic propargyl labeling and sequencing. *Nature Communications,* **14**(1), 7154.

[7] Peng, Y., Meng, H., Ge, R., Liu, S., Chen, M., He, C., and Hu, L. (2022) Detection of m6A RNA modifications at single-nucleotide resolution using m6A-selective allyl chemical labeling and sequencing. *STAR Protocols,* **3**(4), 101677.

[8] Li, Y., Yi, Y., Gao, X., Wang, X., Zhao, D., Wang, R., Zhang, L.-S., Gao, B., Zhang, Y., Zhang, L., Cao, Q., and Chen, K. (June, 2024) 2'-O-methylation at internal sites on mRNA promotes mRNA stability. *Molecular Cell,* **84**(12), 2320–2336.e6.

[9] Tavakoli, S., Nabizadeh, M., Makhamreh, A., Gamper, H., McCormick, C. A., Rezapour, N. K., Hou, Y.-M., Wanunu, M., and Rouhanifard, S. H. (Jan, 2023) Semi-quantitative detection of pseudouridine modifications and type I/II hypermodifications in human mRNAs using direct long-read sequencing. *Nature Communications,* **14**(1), 334.

[10] Chen, L., Zhang, L.-S., Ye, C., Zhou, H., Liu, B., Gao, B., Deng, Z., Zhao, C., He, C., and Dickinson, B. C. (September, 2023) Nm-Mut-seq: a base-resolution quantitative method for mapping transcriptome-wide 2'-O-methylation. *Cell Research,* **33**(9), 727–730.

[11] Xiao, Y.-L., Liu, S., Ge, R., Wu, Y., He, C., Chen, M., and Tang, W. (July, 2023) Transcriptome-wide profiling and quantification of N,6-methyladenosine by enzyme-assisted adenosine deamination.. *Nature biotechnology,* **41**, 993–1003.

[12] Pratanwanich, P. N., Yao, F., Chen, Y., Koh, C. W. Q., Wan, Y. K., Hendra, C., Poon, P., Goh, Y. T., Yap, P. M. L., Chooi, J. Y., Chng, W. J., Ng, S. B., Thiery, A., Goh, W. S. S., and Göke, J. (Nov, 2021) Identification of differential RNA modifications from nanopore direct RNA sequencing with xPore. *Nature Biotechnology,* **39**(11), 1394–1402.

[13] Zhang, M., Jiang, Z., Ma, Y., Liu, W., Zhuang, Y., Lu, B., Li, K., Peng, J., and Yi, C. (October, 2023) Quantitative profiling of pseudouridylation landscape in the human transcriptome.. *Nature chemical biology,* **19**, 1185–1195.

[14] Chan, A., Naarmann-de Vries, I. S., Scheitl, C. P. M., Höbartner, C., and Dieterich, C. (Apr, 2024) Detecting m6A at single-molecular resolution via direct RNA sequencing and realistic training data. *Nature Communications,* **15**(1), 3323.

[15] Zhang, L.-S., Liu, C., Ma, H., Dai, Q., Sun, H.-L., Luo, G., Zhang, Z., Zhang, L., Hu, L., Dong, X., and He, C. Transcriptome-wide Mapping of Internal N7-Methylguanosine Methylome in Mammalian mRNA. *Molecular Cell,* **74**(6), 1304–1316.e8.

[16] Barrett, T., Wilhite, S. E., Ledoux, P., Evangelista, C., Kim, I. F., Tomashevsky, M., Marshall, K. A., Phillippy, K. H., Sherman, P. M., Holko, M., Yefanov, A., Lee, H., Zhang, N., Robertson, C. L., Serova, N., Davis, S., and Soboleva, A. (January, 2013) NCBI GEO: archive for functional genomics data sets–update. *Nucleic Acids Research,* **41**(Database issue), D991–995.

[17] Zhao, H., Sun, Z., Wang, J., Huang, H., Kocher, J.-P., and Wang, L. (12, 2013) CrossMap: a versatile tool for coordinate conversion between genome assemblies. *Bioinformatics,* **30**(7), 1006–1007.

[18] Cappannini, A., Ray, A., Purta, E., Mukherjee, S., Boccaletto, P., Moafinejad, S. N., Lechner, A., Barchet, C., Klaholz, B. P., Stefaniak, F., and Bujnicki, J. M. (January, 2024) MODOMICS: a database of RNA modifications and related information. 2023 update. *Nucleic Acids Research,* **52**(D1), D239–D244.

[19] Yang, X., Yang, Y., Sun, B.-F., Chen, Y.-S., Xu, J.-W., Lai, W.-Y., Li, A., Wang, X., Bhattarai, D. P., Xiao, W., Sun, H.-Y., Zhu, Q., Ma, H.-L., Adhikari, S., Sun, M., Hao, Y.-J., Zhang, B., Huang, C.-M., Huang, N., Jiang, G.-B., Zhao, Y.-L., Wang, H.-L., Sun, Y.-P., and Yang, Y.-G. (May, 2017) 5-methylcytosine promotes mRNA export - NSUN2 as the methyltransferase and ALYREF as an m5C reader. *Cell Research,* **27**(5), 606–625.

[20] Yang, X.-J., Zhu, H., Mu, S.-R., Wei, W.-J., Yuan, X., Wang, M., Liu, Y., Hui, J., and Huang, Y. (July, 2019) Crystal structure of a Y-box binding protein 1 (YB-1)-RNA complex reveals key features and residues interacting with RNA. *The Journal of Biological Chemistry,* **294**(28), 10998–11010.

[21] Quinlan, A. R. (September, 2014) BEDTools: The Swiss-Army Tool for Genome Feature Analysis. *Current Protocols in Bioinformatics,* **47**, 11.12.1–34.
